# Panoramic Two-Photon Imaging and Quantitative Analysis of the Corneal Sub-Basal Nerve Plexus

**DOI:** 10.1016/j.xops.2026.101365

**Published:** 2026-08-17

**Authors:** Wenxin Zhao, Zhen Zhang, Ruijie Wang, Yaguang Hu, Ming Zhang, Yue Zhang, Mengxi Li, Chen Bai, Yanlong Yang, Li Li

**Affiliations:** 1 Department of Ophthalmology, The First Affiliated Hospital of Xi'an Jiaotong University, Xi'an, P.R. China; 2 School of Automation Science and Engineering, Xi'an Jiaotong University, Xi'an, Shaanxi, P.R. China; 3 Shanghai Key Laboratory of Crime Scene Evidence, Shanghai Research Institute of Criminal Science and Technology, Shanghai, P.R. China; 4 State Key Laboratory of Transient Optics and Photonics, Xi’an Institute of Optics and Precision Mechanics, Chinese Academy of Sciences, Xi'an, China

**Keywords:** Two-photon microscopy, Panoramic imaging, Corneal sub-basal nerve plexus, Quantitative analysis, Exvivo imaging

## Abstract

**Purpose:**

Comprehensive and distortion-free mapping of the entire corneal sub-basal nerve plexus (SBNP) remains a major technical challenge, as standard methods are compromised by limited fields of view (e.g., in vivo confocal microscopy) or architectural flattening (e.g., ex vivo flat-mounts). We aimed to develop and validate an integrated imaging-computational platform that overcomes these limitations by enabling panoramic, 3-dimensional (3D) visualization and objective quantification of the intact SBNP.

**Design:**

Experimental study.

**Subjects:**

BALB/c mice.

**Methods:**

We developed a novel platform integrating large-field 2-photon microscopy of the intact, fluid-supported murine cornea with a semiautomated quantitative algorithm (LF-CNP Metrics). This system preserves native corneal curvature, enables pan-neuronal labeling (β-III-tubulin), and allows projection-based morphometric analysis across entire sub-basal nerve plexuses. For proof-of-concept validation, we applied it to mice subjected to in vivo desiccating stress. Animals were assessed at 1, 2, and 4 weeks postintervention; at each time point, clinical parameters (blink rate, tear secretion, corneal fluorescein staining) were measured, followed by the quantification of ten nerve metrics. Associations between nerve parameters and clinical scores were evaluated using Spearman correlation.

**Main Outcome Measures:**

Corneal nerve fiber length (CNFL), density (CNFD), width, branching densities (CNBD, CTBD), arc-to-chord ratio, total curvature, total squared curvature, directional change count, and inflection count metric.

**Results:**

The platform generated panoramic 3D reconstructions of the sub-basal nerve plexus in ex vivo corneal samples, enabling unbiased quantification. Compared to controls, samples from the 1- and 2-week time points exhibited a trend toward reduction in key parameters including CNFL, CNFD, CNBD, CTBD, total curvature, total squared curvature, and absolute direction angle (all η^2^ > 0.14). In the 4-week group, these metrics increased relative to the earlier time points. The corneal fluorescein staining score was negatively correlated with the corneal nerve parameters CNBD, CNFD, CTBD, and the arc length over chord length ratio, with correlation coefficients of –0.74, –0.55, –0.75, and –0.66, respectively.

**Conclusions:**

We present a panoramic imaging and analysis platform that overcomes the critical limitations of field-of-view and tissue distortion, enabling comprehensive and unbiased assessment of the corneal sub-basal nerve plexus for research into neuropathic changes.

**Financial Disclosure(s):**

The authors have no proprietary or commercial interest in any materials discussed in this article.

The corneal nerve, which possesses an innervation density 300–600 times greater than that of the skin, is essential for maintaining ocular surface integrity and function.^1^, ^2^, ^3^ Its precise morphometric assessment is critical, not only for ocular surface diseases like dry eye disease but also as a potential biomarker for peripheral neuropathies.^4^, ^5^, ^6^ However, existing methodologies for comprehensive corneal nerve mapping are fundamentally limited by a trade-off between field of view, architectural fidelity, and practical applicability.

In vivo confocal microscopy (IVCM), the clinical gold standard, is limited by its restricted field of view, hindering comprehensive pan-corneal analysis and longitudinal tracking of widespread pathological changes.^7^ Although image stitching in patients is possible, it is prohibitively slow and offers a limited expanded view. Crucially, these in vivo stitching methods are ill-suited for murine models, where the cornea's minute size and pronounced curvature render precise registration mechanically challenging and prone to deformation. Ex vivo corneal flat-mounting and immunofluorescence staining, the prevailing standard for full-nerve visualization, irrevocably flattens the corneal dome, distorting native 3-dimensional (3D) nerve architecture and compromising accurate morphometry. Recent advances in multiphoton microscopy have enabled impressive in vivo 3D imaging of corneal nerves in transgenic reporter mice.^8^ However, this powerful approach is inherently restricted to genetically labeled subpopulations of nerves (e.g., calcitonin gene‑related peptide-positive fibers), leaving other types of the plexus unvisualized. This highlights a persistent, critical gap: a method capable of mapping the entire corneal nerve network in its authentic 3D state within standard, immunostained samples—the very specimens most widely available in basic research.

Two-photon microscopy is suited to address this gap. It offers a compelling alternative for deep-tissue imaging, characterized by superior penetration depth, inherent optical sectioning yielding high resolution, and minimized photobleaching.^9^ These attributes have cemented its role in diverse fields including tumor immunotherapy, gene therapy, stem cell research, and drug screening and evaluation.^10^, ^11^, ^12^, ^13^ Previous studies showed that two-photon microscopy image quality was superior to confocal microscopy because of a slower signal decay and a higher signal to noise ratio in corneal structure observation.^14^ Yet, its potential for large-field, distortion-free, and fully quantitative analysis of the complete nerve plexus in fixed tissue remains unrealized.

Here, we introduced and validated an integrated analytical platform designed to fulfill this need. Our approach synergizes large-field two-photon microscopy with a novel semi-automated 3D quantification algorithm. We specifically demonstrate that this platform enables the preservation of native corneal curvature in fixed, immunofluorescence staining whole mounts, eliminating the architectural distortions of flat-mounting. Through automated tiling of high-resolution z-stacks, we achieve a comprehensive, pan-corneal field of view. Furthermore, by employing pan-neuronal immunolabeling (TUBB3), we visualize and quantify the entire nerve plexus, transcending the selectivity of transgenic models.

To demonstrate the platform's robustness and analytical power, we applied it to a series of murine corneal samples. This work establishes a practical methodology that provides the research community with a tool for unbiased, high-fidelity quantification of corneal nerves, offering potential for investigating neuropathic changes in both experimental models and archival clinical specimens.

## Methods

### Animal Samples for Platform Validation

All procedures strictly adhered to the Association for Research in Vision and Ophthalmology Statement and were approved by the Animal Ethics Committee of the College of Medicine, Xi’an Jiaotong University. To validate our platform’s sensitivity in detecting neuropathic changes, a proof-of-concept study was performed using corneal samples obtained from a controlled desiccation stress paradigm. Male BALB/c mice (6–8 weeks old), a strain utilized in similar desiccation studies,^15^ were randomly assigned to 4 groups: an untreated control (T0) and groups subjected to in vivo desiccating stress for 1, 2, or 4 weeks (T1, T2, T4). The stress was induced by exposure to a directed airflow (4 m/s, 5 h/day) in a controlled environment (temperature: 20 ± 2°C; humidity: 65 ± 5%). These parameters were continuously recorded and maintained stable. Following each time point, mice were euthanized, and corneas were immediately harvested for subsequent ex vivo imaging and morphometric analysis using our integrated platform.

### Antibodies

Rabbit monoclonal anti-βIII-tubulin antibody (1:200 dilution; ab215037) was purchased from Abcam. Goat anti-rat IgG (H + L) cross-adsorbed secondary antibody, cyanine3 (1:200 dilution; A10522) was purchased from Thermo Fisher Scientific.

### Ocular Surface Parameters Measurement

Blink rate: this was measured for each eye for 1 min by the same person, and the procedure was repeated 3 times.

Quantity of aqueous tear secretion: this was measured by placing phenol red–impregnated cotton threads at the lateral canthus for 60 seconds without anesthesia, and the wetting length was measured using a ruler.

Corneal fluorescein staining score: fluorescein sodium was used for corneal staining, followed by corneal imaging under cobalt blue light. The fluorescein staining score was determined by a masked observer according to the following criteria: (0: no staining; 1: <5 stain points; 2: 5 – 30 stain points; 3: >30 stain points). The final score was the sum of the 4 quadrants. The assessments were performed by a blinded investigator using established methodology according to the previous study.^16^

### Immunofluorescence Staining and Imaging In Situ

Mice were euthanized and the corneas dissected and fixed in freshly prepared 4% paraformaldehyde for 1 hour. Corneas were washed with 0.1 M phosphate‑buffered saline, permeabilized with 1% Triton X-100 in hosphate‑buffered saline for 1 hour and blocked for 2 hours with sealing agent. Corneas were then incubated with rabbit monoclonal anti–βIII-tubulin antibody (1:200) for 72 hours at 4°C, followed by secondary antibodies for 24 hours at room temperature. Finally, stained corneas were sandwiched between 1-mm imaging spacers (2 corneas per well) to prevent compression (Fig 1B) and imaged by two-photon microscopy. Three-dimensional images were generated with field-of-view mosaics algorithm.

**Figure 1:**
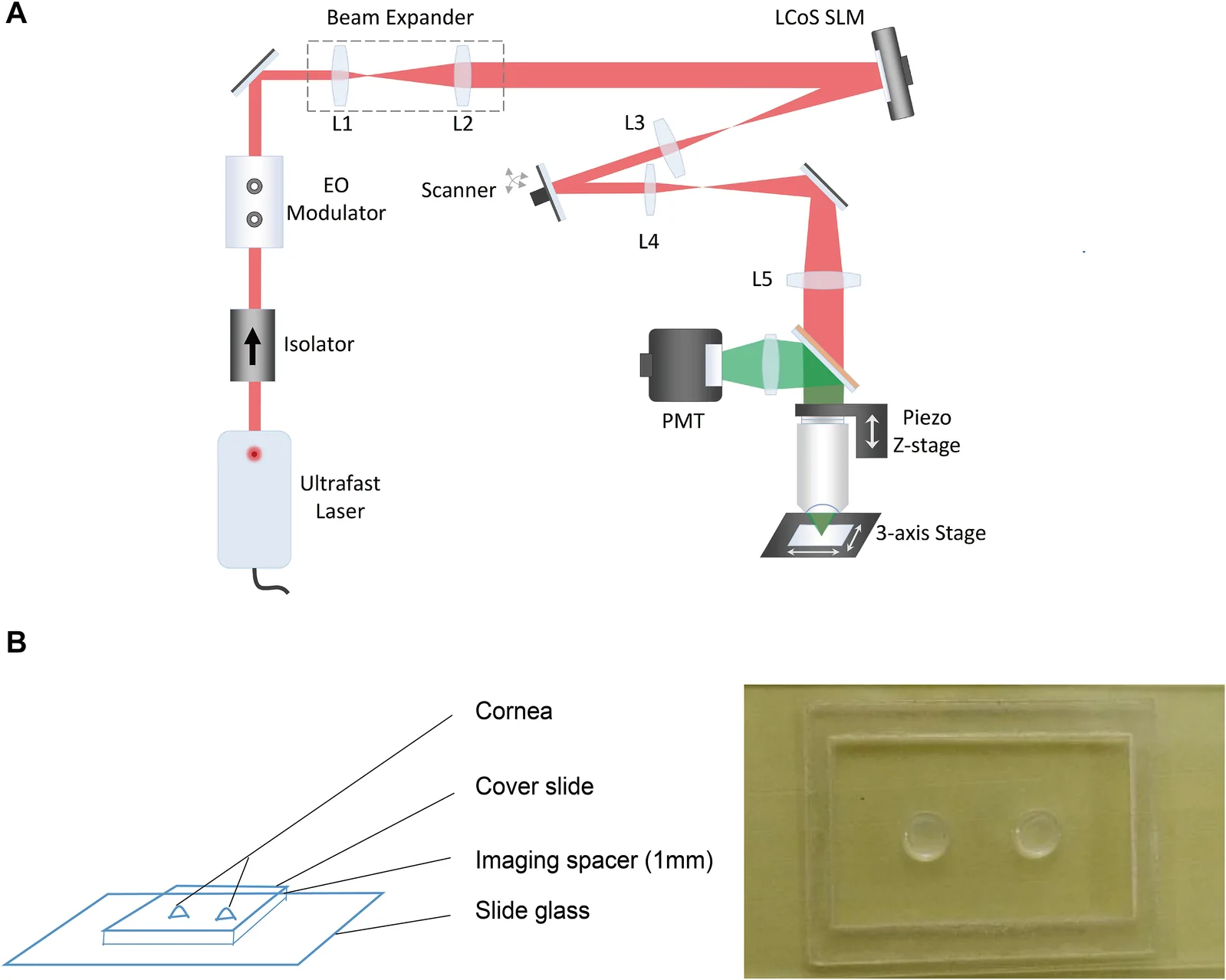
Two-photon microscopy observation and corneal sample preparation. **(A)** Optical path image of two-photon microscopy; **(B)** Imaging spacer with cornea.

### Two-Photon Microscopy System

#### Optical Setup

All images were acquired on a custom-built two-photon laser scanning microscope from Xi’an Institute of Optics and Precision Mechanics, Chinese Academy of Sciences. An ultrafast Ti:Sapphire laser (MaiTai HP, Newport) was tuned to 735 nm for excitation. The beam power was adjusted with an electro-optic modulator (Model 350-80, ConOptics). The beam was expanded before impinging on an LCoS spatial light modulator (X13139-07). With predefined phases, the laser beam was shaped and focused into a Galvo-Resonant Scanner (12 kHz, LSK-GR12, Thorlabs), which can steadily acquire 44 frames per second with 512 × 512 pixels. The emitted fluorescence was collected by a high NA objective lens (N16XLWD-PF 16X, 0.8 NA, Nikon) and detected by a PMT (H7422P-40). The analog control and signal acquisition were composed of NI-PXI modules according to Scan Image recommendations.^17^ A motorized 3-axis stage (MP-285, Sutter Instruments) was used for manually navigating different areas of interest and acquiring image stacks (Fig 1A). Another Piezo z-stage (P-725.4CA, PIFOC, Physik Instrumente) was exploited to rapidly move the objective lens along the z-axis.

### Tile Acquisition and Stitching

To overcome the field-of-view (FOV) limitations of conventional two-photon microscopy (typically 600 × 600 μm^2^) relative to the full murine cornea (≥2000 × 2000 μm^2^, depth 300–600 μm), we upgraded the imaging system to enable automated multi-FOV 3D acquisition and stitching.

### Automated Multi-FOV Acquisition

The imaging volume was defined by manually setting the lateral boundaries (X/Y axes) and depth range (z-axis). The software then automatically calculated the optimal grid of FOVs, including the number of tiles and scanning path, with a preset overlap of 1/10 of the FOV size (typically 512 × 512 pixels per tile) to facilitate subsequent stitching. Within each FOV, z-stack images were acquired by rapid movement of a nanopositioning stage (step size: 2–4 μm; number of slices: 50–300, depending on sample morphology and imaging depth). The motorized stage automatically advanced to the next FOV after completing each tile. For a representative 4 × 4 tile array, total acquisition time for the full 3D volume was approximately 3 minutes.

### Semiautomated 3D Image Stitching

Despite the nanometer precision of the motorized stage, mechanical jitter during acquisition introduces misalignment between adjacent FOVs, rendering simple overlap-based stitching ineffective and prone to artifacts. We therefore developed a semiautomated stitching method. First, all X/Y overlaps were globally adjusted to correct low-order stage drift. Next, users performed fine adjustments for each tile, guided by reference images (either individual slices at selected depths or Z-projections of the entire tile). After alignment, tiles were fused using an alpha-blending method with adjustable parameters to smooth boundaries. Finally, optional denoising and despeckling algorithms were applied to enhance signal-to-noise ratio based on image quality. Overall, the automated portions of the stitching procedure required an average of 15.0 ± 4.3 seconds per sample, with an additional 3 to 5 minutes of manual fine-tuning per data set.

The reconstructed 3D images (Fig 2) provided a comprehensive view of the sub-basal nerve plexus. In the frontal view, both superficial nerve fibers and the deeper nerve trunk were simultaneously visible, allowing assessment of nerve distribution and vortex morphology. Nerve fibers were predominantly distributed in the superior region, while a thickened nerve trunk entered from the corneal periphery and branched progressively toward the central zone. A single vortex structure was observed in the upper central area. The lateral view revealed the z-axis trajectories of nerve fibers and the relative positions of nerve trunks. The dorsal perspective enabled direct visualization of the 3D architecture of nerve trunks, overcoming the limitations of conventional 2-dimensional (2D) fiber analysis. In addition, in the representative video, the 3D images allow for the intuitive observation of all morphological details and changes in the corneal samples, which paves the way for future comprehensive and detailed studies of corneal neuropathies (Supplementary Video 1, available at www.ophthalmologyscience.org).

**Figure 2:**
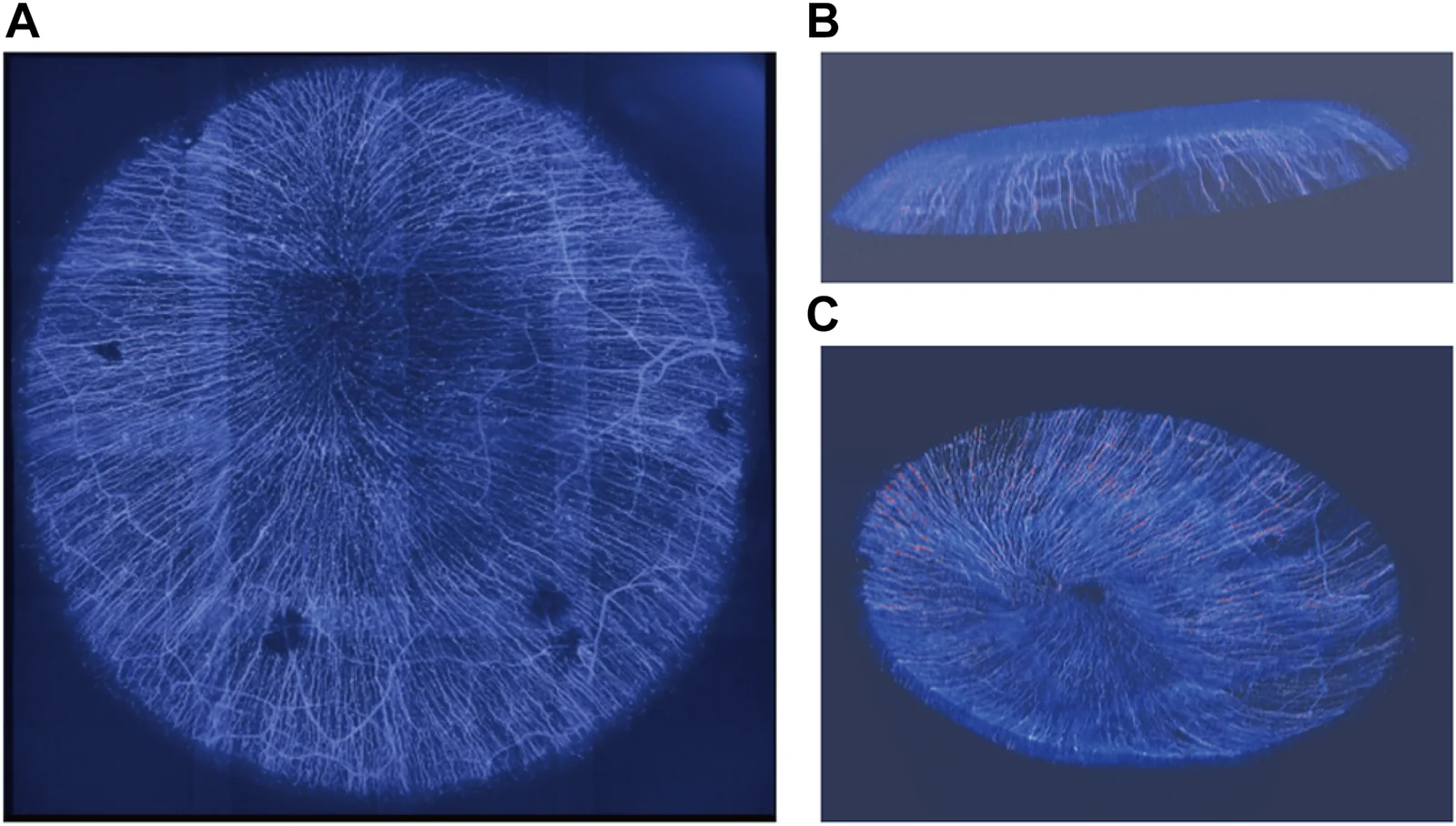
Three-dimensional imaging of the desiccating stress-induced injury murine model acquired by a two-photon laser scanning microscope. **(A)** Frontal view; **(B)** Lateral view; **(C)** Dorsal view.

#### *Development of theLF-CNPMetrics Algorithm*

To enable the quantitative analysis of large-field, 3D two-photon microscopy images of the entire corneal sub-basal nerve plexus, we developed the LF-CNP Metrics—a semiautomated analysis pipeline. This pipeline addresses the limitations of conventional automated nerve analysis tools (such as ACC Metrics), which are often optimized for smaller, 2D fields of view.

Given the initial lack of large-scale annotated training data specific to our imaging modality, we implemented a validated 2-stage approach to ensure accuracy. To reduce computational complexity and suppress irrelevant structures, a subset of raw TIFF images of the corneal sub-basal nerves was binarized using an adaptive threshold (Otsu’s method). Morphological opening and connected-component filtering were then applied to remove small noisy regions unrelated to nerve fibers. The images were meticulously annotated by a trained researcher using NeuronJ software (National Institutes of Health) to establish a reference standard. Specifically, the researcher labeled a portion of prominent and thicker nerve fibers. These nerves were selected for their high contrast and clear trajectories, providing a robust reference for automatic tracking. The number of seeds was adjusted according to the complexity of the nerve plexus (fewer in sparse areas, and more in dense or tortuous areas).

To automatically extract corneal sub-basal nerve fibers, the multiscale Frangi filtering method based on the Hessian matrix was applied. Since corneal nerves form bright tubular structures, the filter was configured to enhance positive ridges. The Hessian matrix H (x, y, σ) was computed over multiple scales σ∈{0.5,1.0,1.5,2.0,2.5} pixels:

$$H\left(x,y,\sigma \right)=\left[\begin{array}{cc} I_{xx}\left(x,y,\sigma \right) & I_{xy}\left(x,y,\sigma \right)\\ I_{xy}\left(x,y,\sigma \right) & I_{yy}\left(x,y,\sigma \right) \end{array}\right]$$

Its eigenvalues $\lambda_{1}$ and $\lambda_{2}$ (with $\lambda_{1}\geq \lambda_{2}$) were then obtained by eigen decomposition and used to characterize local tubular structures.

The Frangi response for bright tubes is defined as:

$$V\left(x,y,\sigma \right)=\exp\left(–\frac{R_{B}^{2}}{2\beta^{2}}\right)\left(1–e\text{xp}\left(–\frac{S^{2}}{2\gamma^{2}}\right)\right)$$

where $R_{B}=\frac{\left|\lambda_{1}\right|}{\left(\left|\lambda_{2}\right|+\epsilon \right)}$, with $\epsilon =10^{–6}$ to prevent numerical instability. And $S=\sqrt{\lambda_{1}^{2}+\lambda_{2}^{2}}$.

To avoid manual tuning, we use expert annotations for parameter calibration. Let $P_{gt}$ denote the annotated pixels within the denoised mask. The optimal β and γ were selected by maximizing:

$$S_{obj}=\mu \left(V\left(P_{gt}\right)\right)–\mu \left(V\left(P_{bg}\right)\right)$$

where μ(⋅)is the mean response and $P_{bg}$ represents background pixels inside the mask. After experimentation, the Frangi sensitivity parameters were fixed at β = 0.5 and γ = 15.

The optimized Frangi response was further refined using morphological thinning, yielding the centerline topology of the corneal subbasal nerve skeleton.

On this basis, we quantified a comprehensive panel of morphometric parameters, which can be categorized into: (1) basic morphometrics: including corneal nerve fiber length (CNFL), corneal nerve fiber density (CNFD), corneal nerve fiber width (CNFW), corneal nerve branch density (CNBD), and corneal nerve total branch density (CTBD) (where CNFD counts total fiber segments per area, CNBD counts branch points on the main nerve fibers per area, and CTBD counts total branch points, including those on main fibers and all higher-order branches, per area); and (2) multidimensional tortuosity assessment. Corneal nerve fiber length was calculated as the sum of Euclidean distances between consecutive skeleton pixels. Corneal nerve fiber width was estimated as twice the mean Euclidean distance transform value sampled along the skeleton. Bifurcation points (pixels with connectivity degree ≥3) were identified to compute CNBD and CTBD. All linear measurements (CNFL, CNFW) were converted from pixels to physical units using the camera calibration factor of 1.1 μm/pixel provided by the confocal microscope manufacturer. For tortuosity, we integrated 5 distinct metrics (arc-to-chord ratio, total curvature, total squared curvature, directional change count, and inflection count metric) to capture different aspects of nerve fiber complexity, moving beyond single-metric evaluations. The arc-to-chord ratio was calculated as the average ratio of the arc length to chord length per nerve fiber. Total curvature and total squared curvature were derived from the integral of curvature and the integral of squared curvature along each fiber, respectively. The absolute directional change count was incremented when the angle between successive virtual segments (connecting the current pixel to the fifth pixel ahead) exceeded 15°. The inflection count metric was defined as the product of (absolute directional change count + 1) and the arc-to-chord ratio, thereby integrating both length and curvature components into the tortuosity assessment. The definitions and formulas for all parameters were based on established methodologies.^18^

### Statistical Analysis

All data were reported as the mean ± standard deviation of triplicate experiments. Given the preliminary nature and limited sample size (n = 3 per group) of this technical validation study, our analysis focused on quantifying the strength of observed trends rather than on conventional significance testing. For clinical parameters (blink rate, tear secretion, and corneal fluorescein staining score) and each of the ten independent neural parameters, differences among the 4 time points were analyzed using the Kruskal–Wallis test. If the overall test indicated a significant (*P* value <0.05), post hoc pairwise comparisons were conducted using Dunn‘s test with appropriate adjustment for multiple comparisons. The magnitude of each temporal trend was further quantified by calculating the effect size (Eta-squared, η^2^). Effect sizes were interpreted using the following benchmarks for η^2^: ≥0.01 (small), ≥0.06 (medium), ≥0.14 (large).^19^ Spearman correlation analysis was used to evaluate the correlation between ocular surface parameters and corneal nerve parameters. Each experiment was evaluated statistically with the GraphPad Prism software package (GraphPad Prism version 8.0). *P* values ˂0.05 was considered to be statistically significant.

## Results

### Comparison of Image Identification Performance between LF-CNP Metrics and ACC Metrics

Given the absence of a gold standard for quantifying panoramic two-photon corneal nerve images in mice, we developed a novel semiautomated algorithm, LF-CNP Metrics, and compared its identification performance against that of the conventional ACC Metrics. Figure 3 illustrates the comparative results. For each figure, Figure 3A shows the original corneal nerve images acquired from two-photon microscopy of dry-eye mouse corneas; Figure 3B displays the nerve fiber skeletons semiautomatically traced by a trained researcher using Neuron J software; Figure 3C shows the corresponding images automatically identified by ACC Metrics (depicted as blue curves) without any manual intervention; and Figure 3D presents the corresponding fiber network identified by LF-CNP Metrics, which incorporates prior knowledge from manual annotation.

**Figure 3:**
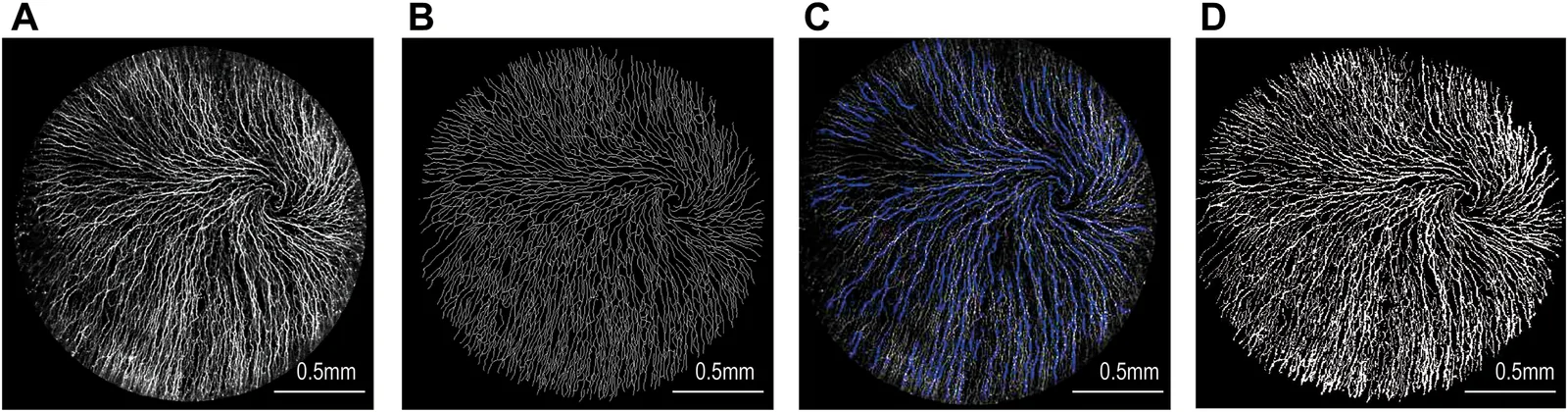
Comparison of image identification performance between LF-CNP metrics and ACC metrics. **(A)** Original corneal nerve images acquired from two-photon microscopy of dry-eye mouse corneas; **(B)** Nerve fibre skeletons semiautomatically traced by a trained researcher using Neuron J software; **(C)** Corresponding images automatically identified by ACC metrics (depicted as blue curves); **(D)** Corresponding fiber network identified by LF-CNP metrics, which incorporates prior knowledge from manual annotation.

### Analysis of Ocular Surface Parameters

Our corneal imaging coupled with automated algorithm analysis effectively detected and robustly quantified the sub-basal corneal nerve damage elicited by the desiccating stress in the mouse model. At baseline, the eye blink rate of untreated (T0) mice was 6.11 ± 2.44/min. Following airflow exposure of 1 (T1), 2 (T2), or 4 weeks (T4), mice displayed reduced blink rates of 4.00 ± 1.44/min (T1), 3.44 ± 2.20/min (T2), and 2.11 ± 0.42/min (T4), respectively (Fig 4A). However, this decreasing trend was not statistically significant (*P* = 0.244). Measurement of aqueous tear secretion (Fig 4B) showed that the T2 group (3.70 ± 1.19 mm/min) produced the highest level, which was higher than the T1 (2.37 ± 0.99 mm/min) and T4 (1.57 ± 0.40 mm/min) groups, and slightly higher than the control (T0, 3.07 ± 0.86 mm/min). However, there was no significant trend among the 4 groups (*P* = 0.185). For corneal fluorescein staining, the Kruskal–Wallis test indicated a significant overall change over time (H = 8.927, *P* = 0.004), with a large effect size (η^2^ = 0.741). Post hoc pairwise comparisons with adjustment for multiple comparisons did not yield statistically significant differences between specific weeks. However, visual inspection of the individual data trajectories revealed a pattern: an increase in staining intensity at T1 (10.00 ± 0.82) and T2 (8.67 ± 1.70) compared to T0 (1.00 ± 0.82), followed by a clear reduction at T4 (2.33 ± 1.25) (Fig 4, *C* and *D*).

**Figure 4:**
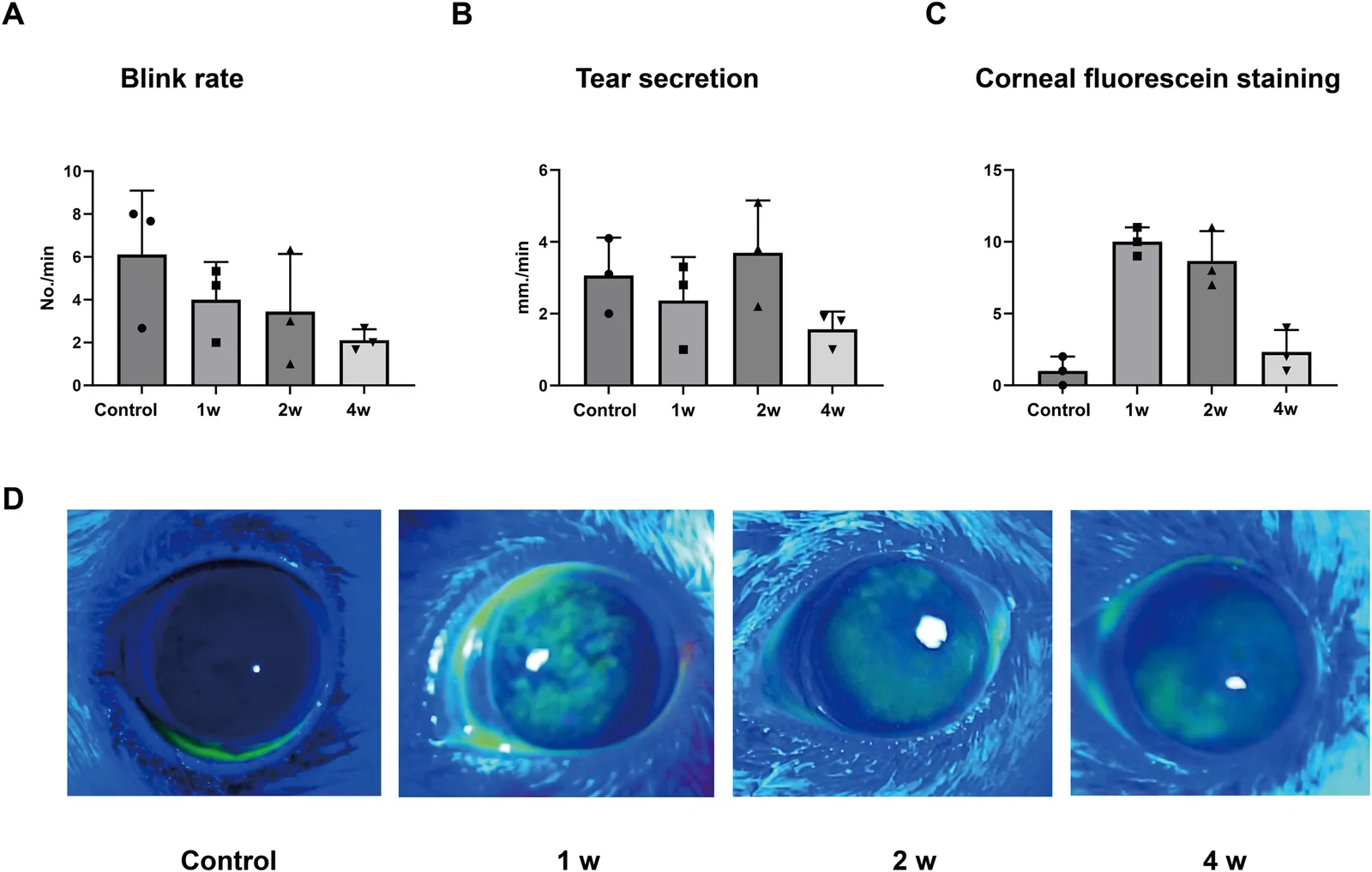
The ocular surface parameters of mouse model. **(A)** Blink rate; **(B)** Aqueous tear secretion; **(C)** corneal fluorescein staining scores; **(D)** Images of corneal fluorescein staining.

### Morphological Changes in the Sub-Basal Nerves

To evaluate the morphological changes in the sub-basal nerves, we used two-photon microscopy to acquire 2D and 3D images of the total corneal nerve. The 2D image is generated by superimposing scan image information captured at intervals of 2 μm corneal thickness. As shown in Figure 5, the desiccating stress induction significantly changes the corneal nerve density and curvature. The density of nerve fibers was reduced at T1 compared to control and further reduced at T2. By week 4 (T4), higher nerve density was observed than at T1 and T2. As for the curvature of the corneal nerves, the images showed that the nerve fibers were straighter in the T1 and T2 groups than in the control, and the disordered, tortuous nerves were seen in the T4 group.

**Figure 5:**
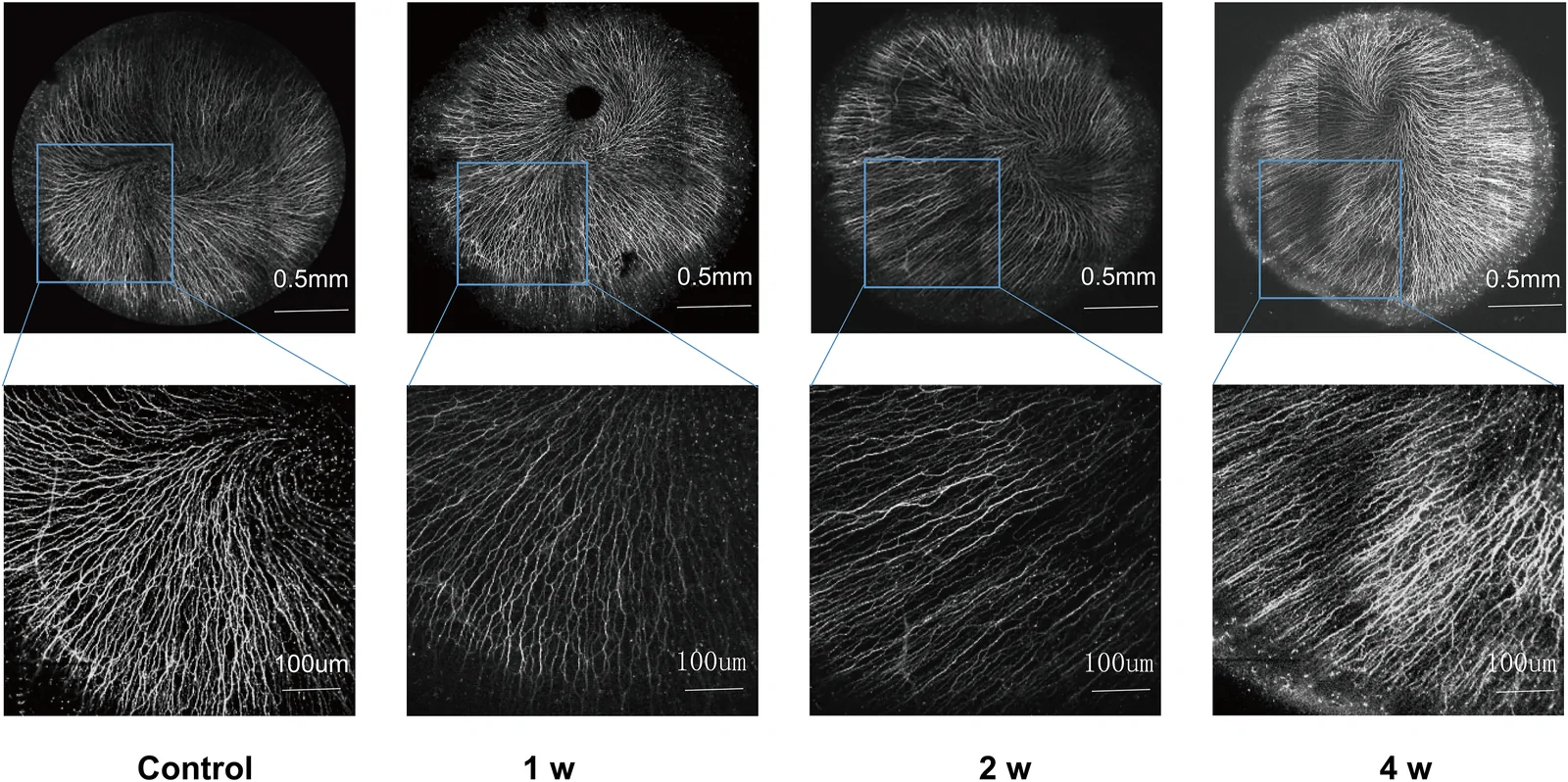
Two-dimensional images of two-photon corneal neuroimaging.

Compared with the control group, the nerve density and curvature decreased in the 1-week and 2-week groups, while the nerve density and curvature increased in the 4-week group compared with the 1-week and 2-week groups. The blue rectangle indicates the representative area.

The 3D images were generated by superimposing single-layer scans in the original orientation to cover a 200-300 μm depth of the cornea, allowing the relative position of the corneal nerve, the complete corneal nerve plexus, and the nerve trunk to be observed (Fig 6). Thus, unlike 2D images, the overall distribution of nerve fibers and their rotational direction, the relative position of the vortex structure, and most importantly, the nerve fibers and nerve trunk can be observed simultaneously. Compared with the control, density of the nerve fibers in the T1 and T2 groups was decreased, and a compensatory increase in curvature was observed in some areas of the T4 group. However, it is difficult to evaluate the corneal nerve fiber morphology accurately because of the large amount of information contained in 3D images. So, our next step was to perform quantitative analysis.

**Figure 6:**
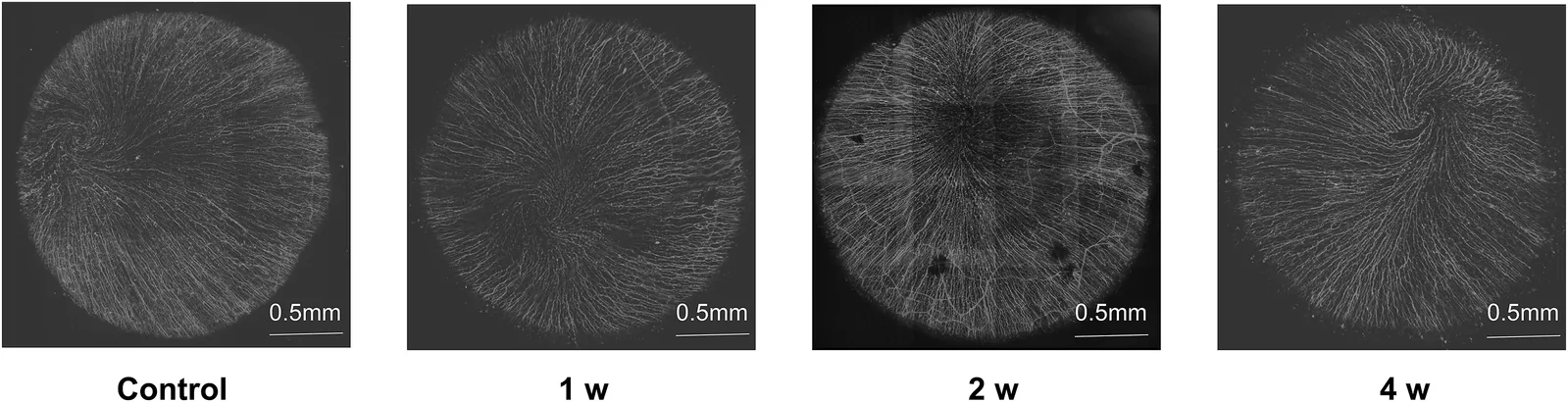
Three-dimensional images of two-photon corneal neuroimaging. The overall distribution of nerve fibers was shown. Nerve fibers and nerve trunk can be observed simultaneously.

### Quantitative Analysis of Changes in the Sub-Basal Nerves

#### Statistical Outcomes of Nerve Parameters

LF-CNP Metrics quantified corneal nerve parameters, including CNFL, CNFD, CNFW, CNBD, CTBD, and nerve tortuosity (Fig 7). Kruskal–Wallis tests indicated significant overall temporal changes for several parameters. Post hoc analysis identified 2 significant pairwise differences: CNFD increased at 4 weeks relative to 1 week (adjusted *P* = 0.040), and the absolute direction angle change was higher at 4 weeks than at 2 weeks (adjusted *P* = 0.019).

**Figure 7:**
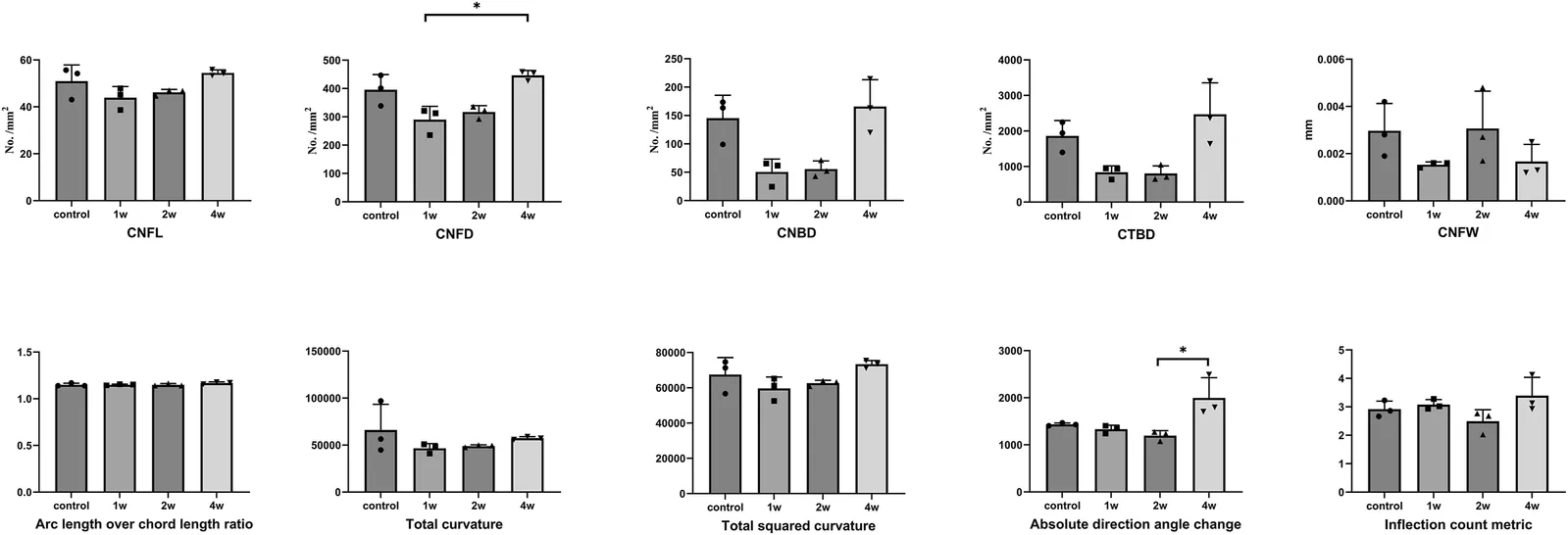
The results of quantitative analysis of corneal nerve parameters by LF-CNP metrics. ∗*P* < 0.05.

The magnitude of change was substantial across parameters. Effect sizes (η^2^) ranged from 0.15 to 0.87, with the largest changes observed in absolute direction angle change (η^2^ = 0.872) and CNFD (η^2^ = 0.782) (Supplementary Table S1).

### Temporal Patterns in Traditional Nerve Metrics

A consistent pattern was observed for key parameters, characterized by a decrease at 1 and 2 weeks compared to control, followed by recovery toward or above baseline by week 4 (Fig 7). For example, CTBD decreased from 1861.8 ± 350.9/mm^2^ at baseline to 839.4 ± 145.7/mm^2^ at week 1, before fully recovering to 2467.8 ± 724.1/mm^2^ at week 4. Similarly, CNFL showed a reduction at week 1 (43.9 ± 3.9 mm/mm^2^) compared to control (51.0 ± 5.6 mm/mm^2^), returning to near-baseline levels by week 4 (54.5 ± 1.1 mm/mm^2^). In contrast, CNFW exhibited a more variable trajectory (Supplementary Table S1, available at www.ophthalmologyscience.org).

### Analysis of Nerve Tortuosity Parameters

Five metrics assessed sub-basal nerve tortuosity. Total curvature and total squared curvature paralleled the trend of CNFL, showing an initial decrease followed by a gradual increase. The absolute direction angle change mirrored the pattern of CTBD, decreasing initially and then rising by week 4. The arc-to-chord length ratio remained stable across all groups. Interestingly, the inflection count metric showed an opposing trend to CNFW, increasing at week 1, decreasing at week 2, and rising again at week 4. Individual data trajectories for all parameters are presented in Figure 7. Detailed descriptive statistics (including mean ± standard deviation for all parameters at each time point) and pairwise comparison results are provided in Supplementary Table S1.

### Correlation Analysis between Ocular Surface and Corneal Nerve Parameters

As the corneal fluorescein staining score was the only significantly altered parameter for the ocular surface, we next determined whether this measurement is correlated with the underlying structural changes observed with two-photon microscopy using the Spearman correlation (Fig 8). The corneal fluorescein staining score was negatively correlated with the corneal nerve parameters CNBD, CNFD, CTBD, and the arc length over chord length ratio, with correlation coefficients of –0.74, –0.55, –0.75, and –0.66, respectively. This indicates that higher fluorescein staining scores are associated with fewer corneal nerve branches and lower nerve density. The corneal nerve parameters were also correlated with each other, but this has little clinical significance since they shared similar calculation methods.

**Figure 8:**
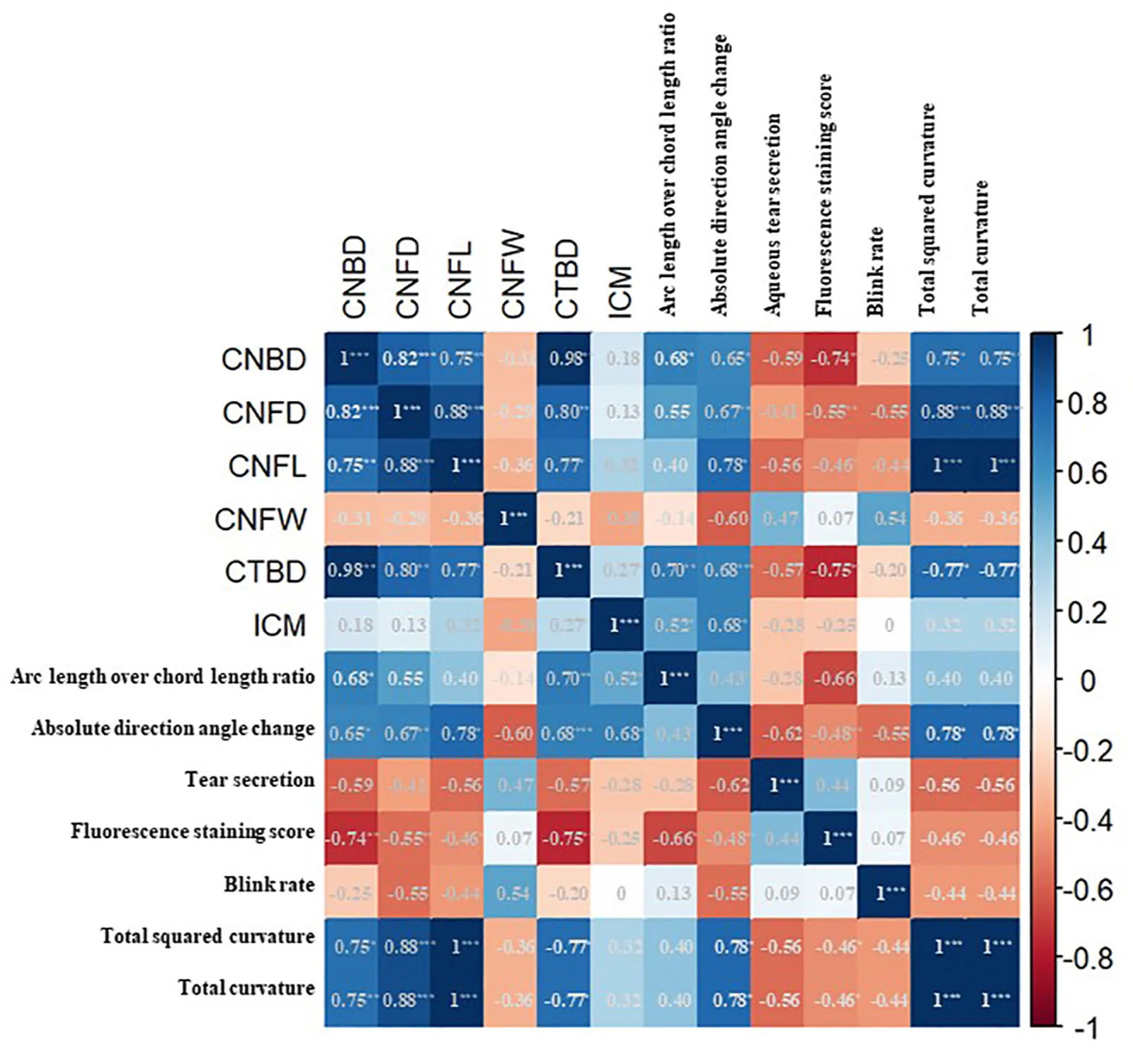
The results of correlation analysis between ocular surface parameters and corneal nerve parameters. ∗*P* < 0.05, ∗∗*P* < 0.01, and ∗∗∗*P* < 0.001.

## Discussion

Although two-photon microscopy offers superior imaging quality for deep tissue,^14^ its application has been largely confined to qualitative observations or analysis of limited fields in corneal research. To bridge this gap, we developed and validated an integrated analytical platform that synergizes large-FOV, 3D two-photon imaging with a novel semiautomated algorithm (LF-CNP Metrics). This platform is designed to address 2 persistent, interconnected limitations in the field: (1) the tissue distortion and spatial information loss inherent in methods requiring corneal flattening, and (2) the lack of a comprehensive, quantitative tool for analyzing the entire corneal sub-basal nerve plexus in its native geometry. By preserving the intact corneal curvature and employing pan-neuronal labeling, our platform enables, to our knowledge, the first holistic and geometrically accurate morphometric mapping of the complete corneal sub-basal nerve plexus.

In this proof-of-concept validation, our platform, applied to a cross-sectional study design, readily detected a spectrum of significant morphometric alterations in the corneal nerve plexus following desiccating stress. The key finding is that the platform resolved a clear biphasic response—characterized by initial degeneration in parameters like CNFL and CNFD, followed by subsequent recovery—even within the experimental context acknowledged to present a less pronounced phenotypic challenge. Notably, this detection was achieved using the BALB/c strain, which has been reported to be less sensitive to environmental desiccation compared to other common strains like C57BL/6.^20^ The fact that our platform identified significant nerve changes under these conditions underscores its high analytical sensitivity. Furthermore, its capability extended beyond tracking major trends to capturing nuanced, parameter-specific dynamics, such as the distinct fluctuation pattern of CNFW. This multifaceted analytical power mitigates the risk of oversight inherent in methods that rely on limited sampling or a single metric. Finally, the strong correlation between the quantified nerve parameters and conventional clinical scores confirms the biological relevance of the platform’s output, validating its utility for producing meaningful research data.

Our platform’s value is best understood in relation to established and emerging corneal neuroimaging methods. Unlike in vivo confocal microscopy, constrained by a narrow FOV, our platform delivers a panoramic, unbiased map of the entire sub-basal nerve plexus. Compared to the tissue distortion inherent in flat-mounting, it preserves the cornea's native curvature for true 3D morphometry. Crucially, our work establishes a distinct and complementary paradigm to advanced in vivo imaging, such as the elegant study by Komai et al. Their approach excels at longitudinal tracking of specific nerve subtypes (e.g., calcitonin gene‑related peptide-positive fibers), providing dynamic functional insights.^8^ In contrast, our platform is architected to establish the definitive structural baseline of the entire sub-basal nerve architecture. By combining pan-neuronal imaging with our custom algorithm, we extract a rich panel of morphometric parameters that quantify the plexus’s structural state with high fidelity. Thus, while their study excels at answering “how specific nerves change over time,” our platform is architected to answer “what is the precise structural state of the entire network.” This makes our platform uniquely suited for decoding the structural substrates of functional observations, and providing ground-truth validation in standard or complex animal models. It thereby serves not merely as a complement, but as a foundational anatomical framework for integrative corneal neuroscience.

A pivotal innovation is the LF-CNP Metrics algorithm, engineered to overcome the analytical bottleneck for large-scale, distortion-free 3D nerve images. Unlike conventional tools like ACC Metrics—which, despite offering rapid automated analysis,^21^ is incompatible with our panoramic data format—or comprehensive commercial suites like Imaris (employed in studies such as Komai et al for 3D reconstruction and volume analysis),^8^ our algorithm provides an integrated, targeted solution. It is designed to efficiently handle datasets an order of magnitude larger in spatial coverage (spanning several millimeters via dozens of tiles, e.g., ∼16 tiles of 600 × 600 μm each, compared to the 9-tile, ∼554 × 554 μm approach typical for live imaging) and obviates the need for the extensive, iterative manual 3D rendering that characterizes general-purpose software workflows.^8^ The LF-CNP Metrics algorithm delivers 2 key, quantifiable advances: (1) it uniquely enables the quantitative morphometric analysis of these expansive, curvature-preserved corneal nerve panoramas, filling a prior methodological gap; and (2) it achieves exceptional computational efficiency, processing each stitched panoramic dataset in under 10 seconds. This represents a drastic reduction in analysis time compared to the multistep, software-dependent processing (involving manual alignment, surface creation, and filament tracing) required for 3D quantification in studies like Komai et al, shifting the timeline from minutes to seconds. Currently operating in a semi-automated mode, the algorithm’s use of expert-annotated skeletons ensures high measurement fidelity while simultaneously generating the structured training data essential for its future evolution into a fully automated tool via deep learning. This development pathway positions LF-CNP Metrics not merely as an analytical tool, but as a scalable open-science resource specifically tailored for the evolving needs of high-throughput, panoramic corneal neuroimaging.

Our panoramic imaging and quantification platform was applied to a controlled desiccating stress paradigm—a model designed to generate a defined spectrum of corneal nerve alterations for methodological validation, consistent with broader reports linking such alterations to ocular surface pathology.^15^^,^^22^, ^23^, ^24^ Utilizing a cross-sectional design that provides high-fidelity tissue samples optimal for undistorted panoramic imaging, the platform successfully captured a complex, time-resolved morphological signature of the entire sub-basal nerve plexus. The platform resolved a coordinated biphasic response: key architectural parameters (CNFL, CNFD, CNBD, and CTBD) decreased at 1 and 2 weeks postinduction, then recovered or exceeded baseline by week 4—a pattern aligning with reports of nerve injury and compensatory change in advanced animal models,^25^ though such structural recovery may not equate to functional normalization. In contrast to clinical studies reporting increased CNFW in dry eye disease patients,^26^ we found no significant difference, a discrepancy potentially attributable to interspecies variation or methodological differences. Concurrently, our multiparametric analysis of tortuosity (including total curvature, squared curvature, and absolute directional change) revealed a distinct, inverse biphasic trajectory—an initial decrease followed by a gradual increase. This aligns with temporal observations in other models^25^^,^^27^ and supports the hypothesis that reduced tortuosity may reflect acute axonal injury, while increased tortuosity could correlate with regenerative processes.^28^ Crucially, the platform’s output demonstrated biological relevance, as fluorescein staining scores correlated negatively with CNBD, CNFD, CTBD, and a tortuosity (arc length over chord length) ratio. The noted discrepancies with prior clinical studies—such as the finding by Ma et al that tortuosity was not correlated with fluorescein staining in dry eye patients^18^ —are not merely inconsistent data but reveal the methodological constraints of prior approaches. These inconsistencies likely stem from (1) sampling bias from analyzing limited, subjectively selected corneal areas versus our panoramic, unbiased census; and (2) definitional heterogeneity in nerve parameters and calculations versus our standardized, algorithm-defined metrics. Therefore, this study delivers more than a novel observation in a specific model. By enabling the conversion of complex neural architecture into a consistent, multiparametric quantitative language, our platform provides a standardized analytical framework to reconcile discordant literature and establish robust morphometric benchmarks. It directly addresses the field’s core challenge of data comparability, establishing a necessary foundation for generating future, directly comparable datasets across diverse studies and models.

This study has some limitations that outline a clear path for future development. First, while the small sample size limits statistical power, this proof-of-concept study successfully demonstrates the sensitivity of the LF-CNP algorithm, as evidenced by large effect sizes and consistent temporal patterns. These robust preliminary findings provide a clear rationale for future validation in larger cohorts. Second, the platform’s initial validation was performed on ex vivo tissue, which provides the essential high-resolution structural benchmark; its translation to in vivo applications remains a crucial next step for longitudinal functional correlation. On the technical front, the current semiautomated analysis requires manual initialization, a step that ensures accuracy but will be targeted for full automation via deep learning. Finally, although the imaging is 3D, the quantitative analysis is currently based on 2D projections of the sub-basal nerve plexus. The development of true 3D morphometric algorithms represents a significant opportunity to further exploit the rich volumetric data.

In summary, we have developed and validated an integrated panoramic imaging-computational platform that overcomes the critical methodological bottlenecks of FOV limitation and tissue distortion in corneal nerve assessment. By preserving native 3D geometry and enabling unbiased, whole-plexus quantification, this platform provides a robust tool for generating high-fidelity morphometric benchmarks. Its utility in delineating complex nerve alterations and structure–function correlations has been demonstrated in a proof-of-concept study. This methodology establishes a new standard for spatially accurate, comprehensive phenotyping of corneal neuropathy, with direct applicability to basic research.
