# Supplementary material for: Panoramic Two-Photon Imaging and Quantitative Analysis of the Corneal Sub-Basal Nerve Plexus
Source: Ophthalmol Sci. 2026 Aug 17;6(10):101365. doi: 10.1016/j.xops.2026.101365 (PMC13627104; doi:10.1016/j.xops.2026.101365)
Supplement: Supplementary file 1 [file mmc2.docx]

Supplementary Table S1. Quantitative analysis of corneal subbasal nerve changes in a murine model of desiccating stress-induced injury

| Parameters | T0 | T1 | T2 | T4 | K-W  p value | Effect Size (η²) | Key Significant Pairwise Finding (Dunn‘s test) |
| --- | --- | --- | --- | --- | --- | --- | --- |
| CNFL (mm/mm^2^) | 51.0±5.6 | 43.9±3.9 | 46.2±1.0 | 54.5±1.1 | 0.161 | 0.276 | NA |
| CNFD (No./mm^2^) | 395.4±44.1 | 289.6±38.7 | 316.9±18.2 | 446.5±14.0 | 0.002 | 0.782 | T4 > T1 (p=0.040) |
| CNBD (No./mm^2^) | 145.4±32.9 | 50.6±18.5 | 55.5±11.7 | 165.7±38.6 | 0.012 | 0.705 | NA |
| CTBD (No./mm^2^) | 1861.8±350.9 | 839.4±145.7 | 803.4±176.7 | 2467.8±724.1 | 0.008 | 0.705 | NA |
| CNFW (um/mm^2^) | 3.0±0.9 | 1.5±0.1 | 3.1±1.3 | 1.7±0.6 | 0.062 | 0.452 | NA |
| Arc length over chord length ratio | 1.15±0.01 | 1.15±0.01 | 1.15±0.01 | 1.17±0.01 | 0.260 | 0.154 | NA |
| Total curvature | 66038.0±22359.9 | 46594.3±4097.9 | 48927.3±1021.0 | 57293.7±1296.0 | 0.194 | 0.231 | NA |
| Total squared curvature | 67513.3±7835.8 | 59617.7±5300.8 | 62621.3±1305.8 | 73344.3±1726.7 | 0.161 | 0.276 | NA |
| Absolute direction angle change | 1433.3±25.3 | 1332.2±69.6 | 1195.0±86.1 | 1997.0±351.4 | <0.001 | 0.872 | T4 > T2 (p=0.019) |
| Inflection count metric | 2.92±0.23 | 3.08±0.15 | 2.49±0.33 | 3.39±0.52 | 0.097 | 0.385 | NA |

CNFL, corneal nerve fiber length; CNFD, corneal nerve branch density; CNBD, corneal nerve branch density; CTBD, corneal nerve total branch density; CNFW, corneal nerve fiber width.

Statistical comparisons across time points were performed using the Kruskal-Wallis test, followed by Dunn's post hoc test for multiple comparisons.

η² = (H - k + 1) / (n - k)
